# Review of Randomized Controlled Trials Using e-Health Interventions for Patients With Eating Disorders

**DOI:** 10.3389/fpsyt.2020.00568

**Published:** 2020-06-12

**Authors:** Mojtaba Ahmadiankalati, Sabine Steins-Loeber, Georgios Paslakis

**Affiliations:** ^1^Eating Disorders Unit, Toronto General Hospital, University Health Network, Toronto, ON, Canada; ^2^Department of Clinical Psychology and Psychotherapy, University of Bamberg, Bamberg, Germany; ^3^Department of Psychiatry, University of Toronto, Toronto, ON, Canada

**Keywords:** eating disorder, anorexia nervosa, bulimia nervosa, binge eating disorder, telehealth, e-health, randomized controlled trial (RCT), treatment

## Abstract

**Background:**

In a world of technological advancements, electronic devices and services seem to be a promising way to increase patients’ engagement in treatment and to help manage their symptoms. Here, we identified and analyzed the current evidence of RCTs to evaluate the effectiveness and acceptability of e-health interventions in the eating disorder (ED) field.

**Methods:**

We screened an initial cluster of 581 papers. In the end, 12 RCTs in clinical ED cohorts were included.

**Results:**

Some studies were conceived as stand-alone interventions, while others were presented as add-ons to ED-specific treatments. Studies varied in the type of EDs under investigation and in the e-health intervention applied (with vs. without therapist support vs. blended interventions; smartphone- vs. web-based). Only four studies reported explicit acceptability measures. Out of those, two reported high acceptability, one reported low acceptability, and one reported no significant difference in acceptability between groups. Four studies reported higher effectiveness of the e-health intervention compared to the control condition, e.g., reduction in maladaptive eating behaviors. Regarding control groups, three used a wait list design and nine had another kind of intervention (e.g., face-to-face CBT, or treatment as usual) as control.

**Discussion:**

So far, the evidence for acceptability and effectiveness of e-health interventions in EDs is very limited. There is also a lack of studies in older patients, adolescents, men, sexual and ethnic minorities. Shame/stigma is discussed in the context of e-health interventions for EDs. It remains unclear how severity of EDs affects the effectiveness of e-health interventions, how patients can channel the knowledge they acquire from e-health interventions into their actual behaviors, and how such interventions can better fit the needs of the individual patient to increase acceptability and effectiveness.

## Introduction

Eating disorders (EDs) are very common, with some studies reporting collective prevalence rates for the three most common forms anorexia nervosa (AN), bulimia nervosa (BN), and binge eating disorder (BED) of up to 10% (1). Additionally, high dropout rates during disorder-specific treatments, low remission rates, high relapse rates as well as mortality rates among the highest for psychiatric disorders (2–6), constitute EDs a relevant public health concern. A significant portion of patients with EDs do not receive adequate treatment, a fact pointing towards barriers in accessing care such as lack of training on eating disorders for general practitioners, delay in treatment referral, and long waiting lists (7–9).

In a world of computers and smartphones, it appears logical to reach out to patients *via* electronic devices in efforts to increase their engagement in treatment and provide them with strategies to manage their symptoms. Collectively known as e-health interventions, these interventions include all those that apply any type of information and communication technologies and cover a spatial distance between professional care providers and care recipients (10). They range from Internet-based stand-alone interventions to newer forms of interventions that combine the strengths of face-to-face and Internet approaches, blended interventions. Great numbers of e-health interventions have been found effective in improving health-related outcomes over a great variety of target conditions and patient groups, both with regard to medical and mental health, in adults and adolescents (11–25). Several studies have identified facilitators and barriers in the implementation and sustainability of e-health interventions (26–28). Apart from the obvious benefits, such as ease of use, cost-effectiveness, and the ability to increase access to services, e-health interventions may give rise to issues of data security, privacy, and technical concerns, may limit team-based approaches, dilute boundaries between health care provider and patient, and impede the development of a therapeutic rapport (29, 30). Attitudes of the general population towards e-health interventions are also not uncritical throughout (31).

E-health interventions have also been applied and evaluated in the context of EDs and have proven to be convenient and acceptable among patients, and to decrease ED-related and comorbid symptoms. Self-monitoring functions offer tracking and analysis of ED-related symptoms and hence help to increase patients´ conscious engagement in treatment (32). In their systematic review, Aardoom et al. studied articles published between January 2013 and September 2015 and found that among the existing e-health interventions, Internet-based cognitive behavioral therapy and guided self-help are two of the most effective approaches in reducing eating disorder psychopathology (33). Barakat et al. (34) aimed to determine which components of digital-based self-help interventions are associated with a decline in ED-related pychopathology and found, for example, that the use of different multi-media channels was a beneficial feature of the interventions, while automated feedback was associated with less improvement. In another systematic review, Schlegl et al. indicated that technology-based interventions not only constitute an option to deliver evidence-based treatments to patients, but can also be applied in prevention, and to support the next of kin of patients with eating disorders (35). Nonetheless, superiority of e-health interventions over face-to-face treatments in the context of EDs has also been questioned (36).

The COVID-19 pandemic has unleashed an unprecedented global race to deliver e-health interventions in times where face-to-face interventions ceased to be an option, and might fundamentally change traditional modi operandi of health care delivery. Given the immense developments in the field, a recent review is necessary to provide an overview of the newest e-health interventions. Not only is it important to keep pace with the newest developments and trends in the field and to assess their effectiveness, but it is also crucial to identify barriers in their applicability to help design more appropriate future interventions. The primary objective of the present paper was to identify, summarize, synthesize, and critically evaluate current evidence regarding effectiveness and user experience/acceptability of e-health interventions in the ED field. To do so, only studies in cohorts diagnosed with an ED and only randomized controlled studies were considered in this work -in contrast to previous reviews that had no restrictions in study design of included studies and also included non-clinical cohorts (34, 36).

## Materials and Methods

We used PubMed (pubmed.gov) to find our primary cluster of papers using the following function: (((((((((((((telemedicine) OR tele-medicine) OR telehealth) OR tele-health) OR ehealth) OR e-health) OR app) OR smartphone) OR video) OR videoconference) OR telepsychiatry) OR tele-psychiatry)) AND (((((((eating disorder) OR eating disorders) OR anorexia nervosa) OR bulimia nervosa) OR binge eating disorder) OR Other Specified Feeding and Eating Disroders) OR Avoidant/Restrictive Food Intake Disorder). Titles, abstracts, and if needed, full text of each publication were screened to evaluate relevance for the purpose of this review. We only considered papers published between April 2016 and February 2020, given that other reviews cover the previous time period (33). We included papers that were written in English, included an e-health intervention (i.e., treatment trials), examined only diagnosed clinical cohorts with an ED, used a randomized controlled design, and had at least 10 patients (adolescents or adults) in the experimental group. We also conducted a manual search of reference lists in eligible articles and identified five additionally relevant papers. Only studies that were already published in peer-reviewed journals were included. Neither control conditions nor outcomes were specified or limited in order to present the broad spectrum of currently available RCTs. Data extracted from each paper to construct this review included objectives, study design, sample size, participants’ demographics (age, gender, and ethnicity), type of ED under investigation, description of treatment arms, primary outcomes, and acceptability of intervention.

The quality of studies was assessed by calculating a quality score as proposed by Gordon et al. (37). A total of 9 questions (1. Was a random or pseudo-random sample used? 2. Was the inclusion criteria clearly defined? 3. Were confounding factors identified and control strategies stated? 4. Were outcomes assessed using objective criteria? 5. Was there sufficient description of the groups? 6. Was there sufficient description of withdrawals and drop-outs? 7. Were the methods of statistical analysis described? 8. Was the source of financial support described? 9. Was there a description of investigators and assessors, with possible conflicts of interest)? were answered using “yes,” “no,” or “unknown,” and the quality score was calculated as the ratio [yes/(no + unknown)]. Comparison of calculated scores between studies allows an evaluation of quality. As an estimate of between-group effect size, Cohen’s d is reported -if studies reported Cohen´s d to describe the magnitude of effects. Study selection and ratings of study quality were performed by two independent researchers and differences of opinion between researchers were resolved through consensus. For quality ratings please refer to **Supplemental Table S1**.

## Results

**Figure 1:**
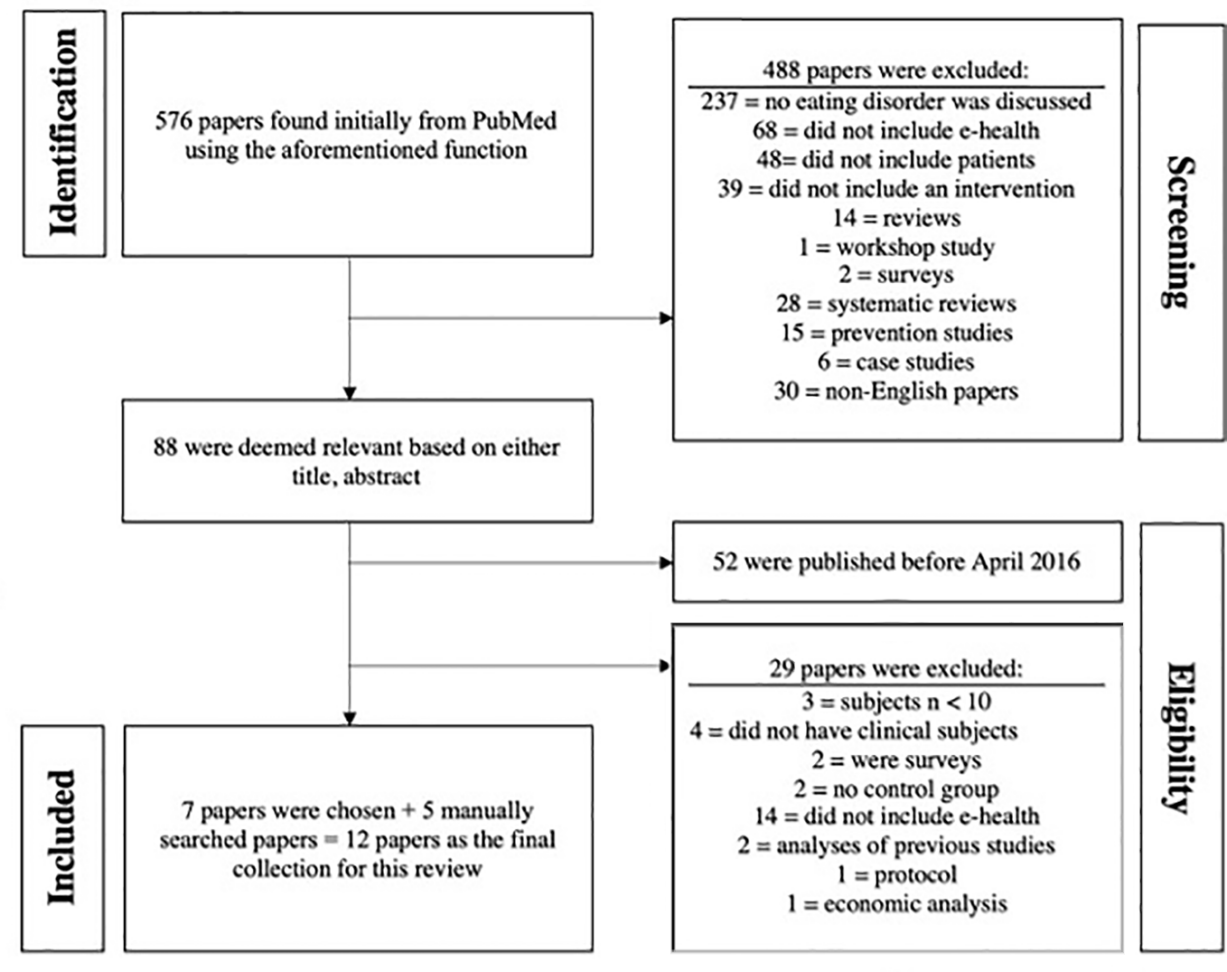
Breakdown of the initial cluster of papers found using the PubMed database.

Initially, 576 papers were found through the PubMed database. Following a screening process based on inclusion criteria and checking the title and abstract of each paper, 488 papers were excluded and 88 were deemed relevant. Out of the 88 papers, 52 were published after April 2016. Out of those 52 papers, 14 did not involve e-health interventions, two were comments on previously published papers, three did not have enough subjects (< 10), two did not have a control condition, two were surveys, four did not include diagnosed clinical ED cohorts, one was a protocol, and one was an economic analysis. We ended up with 7 papers that met our criteria in the end. Five additional papers were found by means of manual search. A total of 12 RCTs were included in this review in the end. Steps of the inclusion process are outlined in **Figure 1** and a brief summary of included papers is found in **Table 1**.

**Table 1:** Summary of RCTs using e-health interventions to modify eating behaviors in adults and adolescents with EDs.

Author	Study design	Sample	N	Intervention	Post-Intervention Results	Length of follow-up period(s)	Follow-up findings	Acceptability	Quality Score
**E-Health interventions with therapist support**									
Zerwas et al. (38)	RCT	Adults with BN (97.4% women)	179	Internet-based CBT group therapy for BN *via* chat platform as treatment group vs. face-to-face group CBT as control group	Higher abstinence from binge eating and purging in CBTF2F compared to CBT4BN	12 months	CBT4BN, but not CBTF2F, continued to reduce binge eating and purging frequency, leading to no differences in abstinence from bingeing/purging between the two groups	Low acceptability	All yes
Wagner et al. (39)	RCT	Adults with BED (96.4% women)	139	Internet-based cognitive-behavioral intervention for adults with BED + high therapist contact as treatment group versus WL control group	Intervention led to significant reduction in number of OBEs and ED psychopathology	3, 6, and 12 months	Superiority of the intervention remained at all follow-ups	N/A	All yes
de Zwaan et al. (40)	RCT	Adults with BED (87.6% women)	178	Internet-based GSH which offered 11 Internet modules and weekly email contacts as treatment group versus control group which offered 20 individual face-to-face CBT sessions	Face-to-face CBT group was superior in reducing days of OBE	6 months and 1.5 years	Significantly higher reduction in ED psychopathology and higher reduction in OBE days in face-to-face CBT over GSH-I at 6 months, but no differences at 1.5 years	N/A	All yes
Jacobi et al. (41)	RCT	Adult women with BN	253	Web-based 9-month long CBT aftercare program as treatment group vs. TAU as control group	Non-significant abstinence from BN between groups; however, significantly lower vomiting episodes in the treatment group	9 months	No difference in abstinence rates between groups at follow-up; number of vomiting episodes was no longer statistically significant at follow-up	N/A	All yes
Strandskov et al. (42)	RCT	Adults with BN or EDNOS (97% women)	92	ACT-influenced Internet-based CBT on EDs as treatment group vs. WL control group	Significant reduction in ED symptoms and body dissatisfaction in the treatment group	N/A	N/A	N/A	All yes
Hildebrandt et al. (43)	RCT	Men (24.9%) and women (75.1%) with BED or BN	225	Noom monitor app plus CBT-GSH telephone coaching as treatment group. Control group involved standard care which meant unrestricted access to clinical resources (but no ED-specific treatments)	Significant reduction in OBE days in the intervention group at the end of intervention compared to the control condition	26 and 52 weeks follow-up	Higher reduction in OBE days and compensatory behaviors in the intervention group at the 52-weeks follow-up	N/A	All yes
**E-Health interventions without therapist support**									
Green et al. (44)	RCT	Women between 14-52 with AN, BN, BED, or OSFED	82	Expanded online version of the Body Project which offered 8 modules and 15 activities designed to reduce thin-ideal internalization, maladaptive social comparison, and self-objectification *via* dissonance induction. WL control group received assessment only	No significant differences between the groups	2 months	No significant effects	N/A	2
**Blended interventions: E-Health interventions added to traditional therapies (face-to-face group or individual therapy)**									
Mazzeo et al. (45)	RCT	Adolescent girls who met criteria for Loss of Control (LOC)-Eating or BED	45	Self-monitoring text-messaging mobile tool (LIDER-8) added to regular ongoing therapy as treatment group vs. weight management program (2BFit) as control group	No significant differences between groups regarding outcome	3 months	No significant difference between groups regarding outcome	High acceptability in both groups. Texting was not well accepted	8
Hildebrandt et al. (46)	RCT	Men (16.7%) and women (83.3%) with BED or BN	66	Noom Monitor smartphone app + conventional CBT-GSH vs. face-to-face CBT-GSH as control group over 12 weeks	CBT-GSH + Noom group reported a greater reduction in objective binge eating episodes at 12 weeks	6 months	Treatment effects were not sustained	N/A	All yes
Cardi et al. (47)	RCT	Patients > 16 with AN (96.8% women)	187	Online workbook consisting of short vodcasts and six 1-h text chat sessions with a recovery mentor + TAU (“Recovery MANTRA” package). Control group received TAU which consisted of outpatient psychotherapy	No significant differences between groups regarding BMI, ED symptomatology, and psychological distress outcomes	6 and 12 months	No significant differences between groups	N/A	All yes
Neumayr et al. (48)	Pilot RCT	Adolescent and adult girls/women with AN	40	8-week smartphone app aftercare intervention with therapist feedback + TAU as treatment group vs. TAU only as control group	Non-significant differences regarding EDE-Q scores and BMI	6 months	Differential effects were absent	High acceptability	All yes
Keshen et al. (49)	RCT	Women > 17 with AN, BN, OSFED, or unspecified feeding or eating disorder	90	Standard care + Recovery Record smartphone app for self-monitoring vs. standard care + paper-based self-monitoring	No significant group differences in ED symptomatology	3 months	No significant differences in ED symptomatology between the two groups	No significant group difference on acceptability	All yes

ED, eating disorder; EDE-Q, Eating Disorder Examination Questionnaire; BMI, body mass index; RCT, randomized controlled trial; AN, anorexia nervosa; BN, bulimia nervosa; BED, binge eating disorder; OSFED, other specified feeding and eating disorders; CBT, cognitive behavioral therapy; CBT-GSH, cognitive behavioral therapy-guided self-help; N, number of participants; TAU, treatment as usual; WL, waiting list; GSH, guided self-help; BDI, Beck Depression Inventory; OBE, objective binge eating; ACT, acceptance and commitment therapy; CBTF2F, CBT Face-to-Face; CBT4BN, CBT for BN.

### E-Health Interventions With Therapist Support

Zerwas et al. conducted a randomized controlled trial to compare the effectiveness of an Internet-based (online chat) cognitive behavioral group therapy for bulimia nervosa (CBT4BN) to face-to-face cognitive behavioral group therapy (CBTF2F) (38). Adults with DSM-IV diagnosis of BN (n = 179), out of which 97.4% were women, were randomized into either of the two groups. Participants in both groups attended 16 × 90 min group CBT sessions. At the end of the interventions, higher abstinence in binge eating and purging behaviors (defined as 0 episodes in the past 28 days) was observed in CBTF2F compared to CBT4BN (|d| = |-0.18|). However, after one year, this difference diminished and both groups exhibited similar levels of BN-related behaviors (d = 0.07); this was attributed to a continuous reduction in binge eating and purging behaviors in the CBT4BN condition even one year after the intervention. CBT4BC was inferior in terms of acceptance compared to CBTF2F. The authors concluded that Internet-based CBT may be associated with a slower trajectory of changes, but reasons for this observation remain to be elucidated (38).

Wagner et al. conducted a randomized controlled trial to determine the effectiveness of an Internet-based cognitive-behavioral intervention for adults with BED (39). This intervention also aimed to evaluate the stability of treatment results over a period of 12 months. A total of 139 adults with DSM-IV diagnosis of BED, of which 134 were women, were randomized into a 16-week Internet-based cognitive behavioral treatment group with high online therapist contacts (TG, n = 69) and a waiting list group (WL, n=70). At the end of the intervention, number of Objective Binge Episodes (OBEs) was significantly reduced in TG vs. WL (d = 1.02). Superiority of the intervention remained at the 3-month (d = 1.31), 6-month (d = 1.05), and 12-month (d = 1.18) follow-up period. TG also showed a significantly higher reduction in all scores of the Eating Disorders Examination Questionnaire (EDE-Q). Despite superiority of TG to WL in terms of symptom improvements, there was a significantly higher dropout rate in TG compared to WL. There were no explicit acceptability ratings mentioned in this study (39).

de Zwaan et al. (40) conducted a randomized controlled trial to determine how an Internet-based guided self-help would help those with BED to reduce their symptoms compared to a face-to-face CBT method. The authors recruited 178 adults with DSM-IV diagnosis of BED (148 women). Patients were randomly assigned into either the treatment group (n = 158) where they completed 11 Internet modules along with weekly email contacts (GSH-I), or the control group (n = 20) where they received 20 individual face-to-face CBT sessions (CBT). Results indicated that CBT was superior in reducing days of OBE compared to GSH-I at the end of treatment (d = 0.16) as well as at the 6-month, but not the 1.5 years follow-up. CBT was also found to be significantly more successful in reducing ED-related psychopathology at the 6-month follow-up. There were no reports on acceptability of either treatment arm (40).

Jacobi et al. conducted a randomized controlled trial to assess the effects of a web-based 9-month long CBT for BN following inpatient treatment (41). Adult women with DSM-IV diagnosis of BN (n = 253) were randomized into treatment and control group (“treatment as usual”, i.e., assessments without recommendations by the research team). The web-based CBT informed users about healthy exercise, body image, self-esteem, and social skills, and provided users with a log to record symptoms. Clinical psychologists provided feedback to users’ entries and communicated with users individually through 1-h chatting sessions. There were no significant differences in abstinence from BN between treatment (18.9%) and control group (21.4%) at the end of the treatment period. Abstinence rates between treatment and control group were not statistically significant at follow-up (9 months after end of the aftercare program), 22.2% vs. 17.3%, respectively. Frequency of vomiting episodes was significantly lower by 46% in the treatment group compared to the control group at the end of the aftercare program, but this difference did not remain significant at follow-up. The authors speculated that due to high symptom severity in this study, acceptability might have been lower for the web-based aftercare intervention compared to treatment as usual. There were no explicit reports made on acceptability of this online intervention among its users (41).

Strandskov et al. conducted a randomized controlled trial to assess the effects of 8-week Internet-based CBT on EDs influenced by Acceptance and Commitment Therapy (42). Adults with DSM-IV diagnosis of BN or eating disorder not otherwise specified (EDNOS) (n = 92) were recruited for this study; 89 out of 92 were women. Patients were randomized into either the treatment (n = 46) or wait list control group (n = 46). The online intervention involved psychoeducation, monitoring eating, establishing regular eating patterns, addressing body image concerns, and helping users develop skills for willingness to change and develop mindfulness in eating, and also included online therapist support/feedback to users. Compared to the control group, the treatment group showed a significant reduction in ED symptoms based on scores in the EDE-Q (significant differences in global scores, d = 0.54) as well as body dissatisfaction based on the Body Shape Questionnaire (BSQ-8C) (d = 0.48). Acceptability of the intervention was not addressed (42).

Hildebrandt et al. (43) conducted a randomized controlled trial to assess the effects of the Noom Monitor smartphone app along with CBT-GSH telephone coaching (telemedicine sessions) on BED and BN. The Noom Monitor was conceived as a self-monitoring tool for meals and snacks, compensatory behaviors, exercise, body checking, cravings, and weight. Adults with DSM-5 diagnosis of BED or BN (n = 225), out of which 169 were women, were randomized into either Noom Monitor + telemedicine sessions (8 coaching sessions over the telephone, administered by health coaches over 12 weeks) (n = 114) or standard care group (n = 111). At the end of the 12 weeks, patients in the intervention group had significantly lower objective binge days than the control group (2.40 ± 3.64 vs. 6.39 ± 6.85 days, respectively). Participants had access to the intervention and were encouraged to make use of it beyond the 12-week period. At a 9-month follow-up, the Noom Monitor + telephone coaching group experienced significant reductions in their objective binge eating days (|d| = |-1.46|), their compensatory behaviors such as vomiting, use of laxatives, and excessive exercise (|d| = |-0.34|), and higher remission rates (56.7% vs. 30%, respectively). The authors considered their results at 52 weeks as a surrogate measure of high adherence to and acceptability of their intervention, but there were no explicit acceptability ratings (43). This study used the same protocol as a previously published pilot study (46), but was different than the pilot study in that telephone coaching was performed by trained health coaches rather than trained ED professionals.

Taken together, three studies examined patients with BN (38, 41, 42), two studies examined BED (39, 40), and one study included patients with BN or BED (43). Five studies used Internet-based interventions (38–42), and one study used a smartphone-based tool (43). All of the identified studies were performed in adults. Four studies compared their e-health interventions against another type of intervention (e.g., treatment as usual or face-to-face interventions) (38, 40, 41, 43), while two applied a wait list design (39, 42). Three studies showed higher effectiveness of the e-health intervention compared to the control group (39, 42, 43), but two studies showed inferiority of the e-health intervention against the control condition (38, 40), and one study showed no differences in primary outcome between intervention and control group, but improvement in secondary outcomes in the intervention group (41). Acceptability was explicitly assessed only in one study; this study reported low acceptability of the e-health intervention (38). All studies fulfilled all 9 quality criteria as described in *Materials and Methods*.

### E-Health Interventions Without Therapist Support

Green et al. conducted a pilot randomized controlled trial to investigate the effects of an online expanded version of the Body Project program on ED symptoms (44). The Body project was originally an ED prevention program offering group sessions and homework assignments towards decreasing thin-ideal internalization; for the purposes of this study it was modified to include verbal, written, and behavioral exercises (8 modules and 15 activities) in an online format. The hypothesis was that ED symptoms and ED-related risk correlates would significantly decrease as result of the intervention. Adolescent and adult girls/women (n = 82) who met DSM-5 diagnostic criteria of an ED (AN, BN, BED, OSFED) or had subclinical ED symptoms, were randomly divided into the Body Project (n = 46) and waitlist control group where they only received assessment (n = 36). Contrary to the original hypothesis, neither ED symptoms, nor risk correlates such as maladaptive social comparison, negative affect, and trait anxiety were significantly different between the intervention and control group; the authors assumed that negative results might have been due to underpowered samples. There were no reports of acceptability (44).

Taken together, this study was performed in adults with AN, BN, BED, or OSFED and applied an Internet-based approach in a wait list design. This study did not show differences in outcomes between intervention and control group (44). This study fulfilled 6 of 9 quality criteria (quality score: 2).

### Blended Treatments: E-Health Interventions Added to Face-to-Face Group or Individual Therapy

Mazzeo et al. (45) conducted a pilot randomized clinical trial to determine the effectiveness of LIBER8, a Dialectical Behavioral Therapy (DBT)-based face-to-face intervention for BED and Loss-Of-Control (LOC) eating amplified by means of mobile based text-messaging. Adolescent girls (n = 45) who met DSM-4-TR criteria for BED were randomized into either LIBER8 (n=28), or the control group 2BFit (n = 17). In LIBER8, patients engaged in self-monitoring of binge eating and LOC eating behaviors *via* text messaging and received automatic feedback messages; patients were also sent reminders if their daily text messages had not been received. 2BFit was conceived as a face-to-face weight management program in which patients were provided education about healthy nutrition and the importance of physical activity. There were no significant outcome differences found between groups, although both groups led to significant improvements. High levels of satisfaction were reported in both LIBER8 and 2BFit groups regarding helpfulness of materials (76% and 86%, respectively), helpfulness of homework assignments (59% and 83%, respectively), and having fun throughout the study (72% and 81%, respectively). Thus, high acceptability in both groups may be inferred. At the same time, texting was not well received; rates of participation in texting ranged between 12-44% (week 1 to 8) (45).

Hildebrandt et al. conducted a randomized controlled trial and sought to find out the differences in clinical outcomes in ED patients who used either the Noom Monitor app (a smartphone guided self-help (GSH) app) + conventional CBT-GSH vs. conventional CBT-GSH alone (46). Conventional CBT-GSH, which was offered across both groups, involved meeting with a therapist and receiving a self-help manual. Adults with DSM-5 diagnosis of BED or BN (n = 66), of which 16.7% were men, were randomized into two groups of CBT-GSH/Noom Monitor (n = 33) and CBT-GSH alone (n = 33). At 12 weeks, the Noom Monitor + CBT-GSH group reported significantly fewer OBEs than the conventional CBT-GSH group. The same group also showed less subjective binge episodes (SBEs) compared to the control group, but this result was not statistically significant. Treatment groups did not differ in any of the outcomes at the 6-month follow-up. Treatment retention in both groups was found to be similar (78.8%). There were no explicit acceptability ratings (46).

Cardi et al. examined the RecoveryMANTRA online intervention during the early (motivational) phase of AN treatment (47). Adults and adolescents with DSM-5 diagnosis of AN (n = 187), out of which 181 were women, were randomized to receiving either RecoveryMANTRA + treatment as usual (n = 99) or treatment as usual (TAU) only (n = 88). RecoveryMANTRA consisted of a series of workbooks, a collection of short video clips (vodcasts) and 6 × 1-h chat sessions which allowed text messages only. TAU consisted of outpatient psychotherapy. Increase in BMI was the primary outcome. ED symptoms and psychological distress outcomes (depression and anxiety) were also examined. There were no significant differences between intervention and control group with regard to changes in BMI (d = 0.2), or ED-related symptoms, depression, anxiety, and work and social adjustment at any time point. However, patients in the RecoveryMANTRA group exhibited significantly higher confidence in their ability to change and a higher ability to build rapport with their therapist at 6 weeks. No acceptability ratings were reported (47).

Neumayr et al. conducted a 8-week pilot randomized controlled trial to determine effectiveness and acceptability of a smartphone app (German version of “Recovery Record”) which included self-monitoring of eating disordered behaviors and related thoughts and feelings, and also offered therapist feedback to patients with AN (48). The app was conceived as a CBT intervention, but also included elements of DBT, ACT, and MET (Motivational Enhancement Therapy). Feedback was provided twice a week as a post-discharge intervention. Adolescent and adult girls/women who met ICD-10 criteria of AN (n = 40) were randomized into two groups of smartphone-based intervention/therapist feedback + treatment as usual (n = 20) vs. treatment as usual alone (n = 20). Treatment as usual consisted of a customized post-discharge treatment. BMI change following discharge was the primary outcome. The control group experienced a greater, yet non-significant drop in their BMI compared to the treatment group both baseline to post-intervention (|d| = |-0.24|) and baseline to the 6-month follow-up (|d| = |-0.06|). With regard to EDE-Q global scores, there was a non-significant reduction in ED symptoms observed in the intervention group both baseline to post-intervention (d = 0.56) and baseline to the 6 month follow-up (|d| = |-0.11|). High ratings of satisfaction and acceptability for a number of features of the smartphone app postintervention was described (48).

Keshen et al. (49) conducted a randomized controlled trial to compare the effectiveness and acceptability of the Recovery Record app (an electronic self-monitoring tool through which also feedback was provided) vs. paper-based self-monitoring. Adult women with DSM-5 diagnosis of AN, BN, OSFED, or Unspecified Feeding or Eating Disorder (n = 90) were randomly assigned into either standard treatment + Recovery Record or standard treatment + paper-based self-monitoring. Standard treatment included intensive treatment (group and individual therapy and supervised meals) at an outpatient ED clinic for four days a week for up to 32 weeks. There were no significant differences in ED-related symptoms between the two groups over time. There were also no significant group differences observed in terms of acceptability (d = 0.36) between the intervention and control group (49).

Taken together, four studies used a smartphone app (45, 46, 48, 49) and one study applied a combination of Internet-based and smartphone-based intervention (47). Two studies were performed in AN (47, 48), one study in BED and LOC eating (45), one study in AN, BN, and OSFED (49), and one study in BN or BED (46). One study included adolescents only (45), two studies included adolescents and adults (47, 48), and two studies were performed in adults (46, 49). There were no wait list designs; in all of the above-mentioned studies, an e-health intervention was compared against another type of intervention. With regard to effectiveness, four studies showed no differences between e-health intervention and control group in primary outcomes (45, 47–49); only one study showed higher effectiveness of the intervention compared to the control condition (46). High acceptability was explicitly reported in two studies (45, 48), and no difference in acceptability ratings were reported in one study (49). The rest of the studies did not perform explicit acceptability ratings. With the exception of the study by Mazzeo et al. (45) that fulfilled 8 of 9 quality criteria (quality score: 8), all other studies fulfilled all 9 quality criteria described in “methods”.

## Discussion

The purpose of this review was to identify, summarize, synthesize, and critically evaluate current evidence regarding effectiveness and user experience/acceptability of e-health interventions in the ED field. In the end, 12 RCTs using an e-health intervention in clinical ED cohorts were included.

Some studies investigated e-health interventions as an alternative to conventional types of ED treatment (38–43), while others combined an e-health intervention with an ongoing face-to-face treatment (45–49). There were also studies that compared e-health interventions using a wait list design (39, 42, 44).

### Clinical Groups, Types of Interventions, Number of Participants, Demographic Characteristics

The 12 included studies differed in terms of the specific type of EDs under investigation. There were patients suffering from BN (38, 41–44, 46, 49), AN (44, 47–49), BED (39, 40, 43–46), and OSFED (44, 49). Overall, given the high prevalence, morbidity, and mortality of EDs, the evidence on RCTs applying e-health interventions in the field appears rather scarce. The small number of studies per ED entity limits the options of generalizability of effectiveness and acceptability outcomes, as discussed further below.

E-health interventions for patients with EDs provide versatile, elaborate designs, ranging from structured modules and associated activities (44), over vodcasts and chat sessions (47) to regular therapist support online sessions (39). Six studies included web-/Internet-based interventions (38–42, 44), five studies included smartphone-based interventions (43, 45, 46, 48, 49), and one study used a combination of the two (47); this last study included n = 187 participants (47). The number of participants recruited for smartphone app studies varied from 40 to 225, and was below 100 in four out of five. The number of participants recruited for web-based studies varied from 82 to 253, and was above 150 in three out of six studies. Thus, the number of participants recruited for web-based studies was higher than that of smartphone-based studies. Additionally, the types of e-health interventions studied were too heterogeneous in nature to be able to draw conclusions as to whether Internet- or smartphone-based interventions are to be preferred, although single studies have favored smartphone-based solutions because they offer the option of reminders to increase adherence to the intervention (45).

Eight of 12 studies included adult/young adult (> 17 yrs.) cohorts (38–43, 46, 49); three studies used mixed adult/adolescent cohorts (44, 47, 48), and one study was performed in adolescents (45). Except Zerwas et al. (38) who did not provide information about the age of participants, the range of patients in all included studies varied between 13-61. Thus, effectiveness and acceptability of e-health interventions in older cohorts, who might not be as familiar with newer technologies, cannot be determined at present. At the same time, there is dearth of e-health studies in adolescents with EDs.

Participants were all women in five out of the 12 studies included in this review, and for the remaining seven studies, participants were on average 90.5% women, with a minimum of 75.1% (43) and a maximum of 97.4% (38). Conclusions drawn from these studies basically apply to women and cannot be generalized to men with regard to acceptability and effectiveness. Further research on the impacts of e-health interventions on men is warranted, given the fact that treatment outcomes in EDs may be a function of gender (50), and that women and men might differ with regard to preferred treatment interventions (51). The same applies to the necessity to address diverse populations, e.g., non-binary individuals, in future studies.

Six out of the 12 studies in this review did not provide specifics regarding race/ethnicity of their participants. Out of the remaining six, participants were on average 76.1% white/Caucasian with a minimum of 44.4% (45) and a maximum of 97.5% (47). Similar to gender, there is a need to investigate the effectiveness and acceptability of e-health interventions across ethnicities with EDs; race and ethnicity have been described to have an impact on number of ED-related symptoms and their severity, and to be associated with socioeconomic status which is likely to affect the odds of pursuing care (50).

Overall, 10 of 12 studies fulfilled all 9 quality criteria. One study fulfilled 6/9 quality criteria (44) and another fulfilled 8/9 criteria (45).

### Acceptability

Acceptability of e-health interventions was reported explicitly in only a few studies included in this review. Other authors drew indirect conclusions regarding acceptability of their interventions based on drop-out rates and levels of compliance with the proposed programs. Zerwas et al. (38) reported both high engagement failure and low acceptability in participants. Mazzeo et al. (45) and Neumayr et al. (48) reported high acceptability of their proposed interventions. Keshen et al. (49) found no significant differences in acceptability between experimental and control groups. It might be argued that severity of illness and patients´ daily routines have an impact on whether patients are able to fully and ideally comply with the demands of an e-health intervention. Patients with severe EDs might be less inclined to use web-based interventions following discharge, as indicated by Jacobi et al. (41). In contrast to web-based solutions, smartphone app-based interventions have the potential to increase adherence and effectiveness by providing users with reminders that may play a significant role in the build-up of desired behaviors (45), but may result in low acceptability levels, if users find them to be an obstruction to their daily routines, as indicated by Lindgreen et al. (52). To be able to leverage the full potential of these interventions, future endeavors should systematically assess acceptability and focus on ways to increase adherence and acceptability rates.

### Effectiveness

Effectiveness of interventions is best determined by comparing outcomes of e-health interventions to control conditions. Jacobi et al. (41), Green et al. (44), Mazzeo et al. (45), Cardi et al. (47), Neumayr et al. (48), and Keshen et al. (49) could not show any significant differences in ED-related symptom reduction in primary outcomes across e-health intervention and control groups. However, studies might have been in part underpowered as they only included small sample sizes (e.g., n = 45 and n = 40 in the studies by Mazzeo et al. (45) and Neumayr et al. (48), respectively). Zerwas et al. (38) and De Zwaan et al. (40) showed inferiority of the e-health intervention against the control group; this, however, was reflected in statistically significant p-values rather than relevant effect sizes. Hence, it was four studies that reported higher effectiveness of e-health interventions over control conditions in the end (39, 42, 43, 46). For instance, Wagner et al. (39) reported a significant reduction in number of objective binge eating episodes as result of the intervention. Overall, based on the RCT findings presented here, effectiveness of e-health interventions appears to be limited. Specific factors increasing vs. factors diminishing effectiveness should be addressed in future studies.

Aside from Green et al. (44), all other studies included in this review involved a therapist who communicated with participants and provided them with feedback. Neumayr et al. (48) stated that the majority of patients in their study reported that the most helpful feature was the possibility of receiving therapeutic feedback and chatting with an ED expert. Aardoom et al. (53), in a previous study (not included in this review) have shown that the frequency/intensity of therapist support does not significantly alter outcomes but instead increases satisfaction with the intervention (53). The study by Strandskov et al. (42) raised the effectiveness-related important question of why patients who attend Internet-based psychoeducation and obtain a significant amount of information and skills are often not able to implement their knowledge to manage their ED symptoms. The authors suggested that future studies should look into how patients can actually put their skills into practice, which could significantly change both acceptability and effectiveness of e-health interventions designed for patients with EDs.

#### Shame/Stigma and Financial Barriers to Care as Possible Advantages of E-Health Interventions

Zerwas et al. (38) and Wagner et al. (39) interpreted their results in a completely opposite manner. While Zerwas et al. (38) attributed the superiority of face-to-face groups (over Internet-based treatment) in BN to within-group social exposure, Wagner et al. (39) explained the superiority of their online intervention for BED with the degree of shame and stigma patients with BED face, thus preferring to engage in an online intervention than in face-to-face groups. Aardoom et al. (54), in a study that included individuals who self-reported ED symptoms, also identified shame in those reporting symptoms of BN as a possible motivational factor contributing to higher treatment response rates in patients showing bulimic rather than anorectic psychopathology. Green et al. (44) indicated that negative affect, trait anxiety, and maladaptive social comparison are strong correlates of ED-specific psychopathology and also supported the notion that shame and stigma may motivate patients to engage in online interventions and hence achieve positive results. Watson et al. (55) (not included in this review) also speculated that online CBT may be more appropriate for those patients struggling with health care access barriers such as embarrassment, stigma, and social anxiety. At the same time, studies should address the question whether online interventions foster avoidance and might have a negative impact in the long run after all. To support this notion, Neumayr et al. (48) raised the assumption that patients in the online intervention group were probably less likely to pursue any further help, which might have contributed to symptom increase over time. Based on just the limited current data, it is not possible to draw inferences about the influence of feelings of shame upon the success of interventions of the kind, or to conclude whether different types of EDs (e.g., BN vs. BED) experience social exposure in groups differently, thus requiring different types of interventions.

Cost-effectiveness and cost-utility of online ED interventions was explored previously by Watson et al. (55); the authors considered online interventions a viable alternative for patients with EDs who do not have easy access to specialized treatments. Hildebrandt et al. (43) observed that participants with lower household income showed better improvement in depressive symptoms, and suggested that e-health interventions might prove more beneficial for patients who face financial barriers to expert care. Watson et al. (55) speculated that patients with less ability to commit financially to conventional treatment may find online interventions more feasible and hence benefit from these.

### Strengths

All papers included in this review have examined clinical cohorts and used a RCT design. By contrast, previous reviews had no restrictions regarding study design or participants’ clinical status, thus including uncontrolled studies or studies in non- or sub-clinical cohorts (34, 36). The studies in this review examined both adolescents and adults. This review was carried out during the unfolding of the COVID-19 pandemic and is therefore believed to be of great practical benefit for clinicians as it offers new insights on non-face-to-face as well as blended interventions for various EDs.

### Limitations

Based on the present results, no conclusions on acceptability and effectiveness can be drawn regarding symptom severity, as not sufficient studies have examined e-health interventions patients in different stages of their ED. The scarcity of studies in relapse prevention following intensive ED-specific treatment also points towards an increased need to implement and test an aftercare support infrastructure using e-health interventions. Despite following a meticulous search and screening approach, we only searched one database and thus cannot exclude having overseen studies that should have been included in this review. We finally included 12 studies; these were randomized controlled trials and only included clinical ED cohorts. Thus, the current evidence is not broad enough to allow generalized conclusions on acceptability and effectiveness in clinical ED populations.

### Future Directions

This work adds to reviews of evidence on effectiveness and acceptability of e-health interventions in the field of EDs and is especially relevant during the COVID-19 pandemic. The current pandemic has disrupted traditional models of care, has established involuntary self-isolation and physical distancing as the new norm for a thus far undefined period of time, and has activated unprecedented global efforts to adopt virtualized forms of treatments, obviating the need for face-to-face meetings between patients and health care professionals (56). Regulatory waivers have been applied to facilitate rapid adaptations of existing systems of care, and further regulations and rules are to be expected in this regard. E-health interventions bear a great future potential by virtue of their ability to easily bridge spatial and temporal gaps between providers and recipients of health care interventions, but training among health care professionals is a priority (57). Regardless, this potential can only be fully exploited by means of interventions whose effectiveness has been convincingly shown. Also, the question arises whether the currently observed transformation of health care services towards e-health solutions will continue to be in demand once the pandemic has subsided (57). With regard to EDs, our findings show that increasing acceptability of e-health interventions should be more in focus of future research, along with investigation of factors that promote or impede effectiveness. Continuous research on e-health models of care is necessary to make sure that advantages of these models are emphasized, effectiveness is secured, and high acceptability rates warranted.

## Conclusions

We have described the most current evidence and have attempted to point out differences and common denominators between randomized controlled e-health trials in clinical ED cohorts, which could help design and implement future interventions. We could only identify a small number of relevant studies, but a big gap in knowledge with regard to acceptability and effectiveness of e-health interventions, including lack of studies in older patients, in men, in sexual and ethnic minorities. Further research in cohorts with EDs is much needed in this relatively new and quickly evolving field of health care delivery. Next to the need for more studies, the question for more tailored e-health interventions that better fit the needs of individual patients with EDs may also be raised.

## Author Contributions

MA and GP screened for relevant publications. MA wrote the first draft of the paper. GP and SS-L critically revised and edited subsequent versions of the paper.

## Conflict of Interest

The authors declare that the research was conducted in the absence of any commercial or financial relationships that could be construed as a potential conflict of interest.
