# Supplementary material for: Review of Randomized Controlled Trials Using e-Health Interventions for Patients With Eating Disorders
Source: Front Psychiatry. 2020 Jun 12;11:568. doi: 10.3389/fpsyt.2020.00568 (PMC7304304; doi:10.3389/fpsyt.2020.00568)
Supplement: Supplementary file 1 [file Table_1.docx]

| Study | Was a random or pseudo-random sample used? | Were inclusion criteria clearly defined? | Were confounding factors identified and control strategies stated? | Were outcomes assessed using objective criteria? | Was there sufficient description of the groups? | Was there a description of withdrawals and drop-outs? | Were methods of statistical analysis described? | Was the source of financial support described? | Was there a description of investigators and assessors, with possible conflicts of interest? | Total quality score |
| --- | --- | --- | --- | --- | --- | --- | --- | --- | --- | --- |
| Zerwas et al. 2016 (Zerwas et al., 2016) | Y | Y | Y | Y | Y | Y | Y | Y | Y | All Y’s |
| Wagner et al. 2016 (Wagner et al., 2016) | Y | Y | Y | Y | Y | Y | Y | Y | Y | All Y’s |
| de Zwaan et al. 2017 (De Zwaan et al., 2017) | Y | Y | Y | Y | Y | Y | Y | Y | Y | All Y’s |
| Jacobi et al. 2017 (Jacobi et al., 2017) | Y | Y | Y | Y | Y | Y | Y | Y | Y | All Y’s |
| Strandskov et al. 2017 (Strandskov et al., 2017) | Y | Y | Y | Y | Y | Y | Y | Y | Y | All Y’s |
| Hildebrandt et al. 2020 (Hildebrandt et al., 2020) | Y | Y | Y | Y | Y | Y | Y | Y | Y | All Y’s |
| Green et al. 2018 (Green et al., 2018) | Y | Y | N | Y | Y | N | Y | Y | N | 2.00 |
| Mazzeo et al. 2016  (Mazzeo et al., 2016) | Y | Y | N | Y | Y | Y | Y | Y | Y | 8.00 |
| Hildebrandt et al. 2017 (Hildebrandt et al., 2017) | Y | Y | Y | Y | Y | Y | Y | Y | Y | All Y’s |
| Cardi et al. 2019 (Cardi et al., 2019) | Y | Y | Y | Y | Y | Y | Y | Y | Y | All Y’s |
| Neumayr et al. 2019 (Neumayr et al., 2019) | Y | Y | Y | Y | Y | Y | Y | Y | Y | All Y’s |
| Keshen et al. 2020 (Keshen et al., 2020) | Y | Y | Y | Y | Y | Y | Y | Y | Y | All Y’s |

Supplemental table S1: Assessment of quality scores. Y: Yes, N: No, UC: Unknown.

Total score was calculated through the following equation: [(Number of Y’s) / (Number of N’s + Number of UC’s)]
